# Tip-of-the-Tongue and Feeling-of-Knowing Experiences Enhance Metacognitive Sensitivity of Confidence Evaluation of Semantic Memory

**DOI:** 10.5334/joc.442

**Published:** 2025-04-28

**Authors:** Ali Pournaghdali, Bennett L. Schwartz, Fabian A. Soto

**Affiliations:** 1 Leonard Davis School of Gerontology, University of Southern California, US; 2 Department of Psychology, Florida International University, US

**Keywords:** Metacognition, Mathematical Modeling, Memory

## Abstract

In this study, we used a multidimensional extension of signal detection theory called general recognition theory (GRT) to evaluate the influence of tip-of-the-tongue states (TOT) and feeling-of-knowing (FOK) experiences on the metacognitive sensitivity of recognition confidence judgments. In two experiments, we asked participants to recall names of famous individuals (Experiment 1) or to recall correct answers to a series of general-knowledge questions (Experiment 2). If recall failed for any trial, participants provided metacognitive judgments of TOT and FOK, memory recognition responses, and metacognitive judgments of confidence on those recognition responses. To evaluate the influence of TOT and FOK on the metacognitive sensitivity of confidence judgments, we fit two different GRT models and constructed two sensitivity vs. metacognition curves, each representing changes in metacognitive sensitivity of confidence, as a function of the strength of TOT or FOK. The results showed that experiencing a TOT or a high FOK is associated with an increase in metacognitive sensitivity of confidence judgments. These results are the first report of influence of TOT and FOK on metacognitive sensitivity of confidence.

## Introduction

Metacognition is broadly defined as the ability to monitor, evaluate, and control our cognitive and perceptual processes (Koriat 2007; Metcalfe & Shimamura 1994) and is largely measured via asking people to report different metacognitive judgments, such as feeling-of-knowing (FOK; Koriat 2000; Metcalfe 1986; Schwartz & Metcalfe 1992), tip-of-the-tongue states (TOT; Schwartz & Cleary 2016; Schwartz & Pournaghdali 2020), judgments-of-learning (JOL; Bjork, Dunlosky, & Kornell 2013; Nelson & Dunlosky 1992), and post-decision confidence (Grimaldi, Lau & Basso 2015; Rahnev et al. 2020). A major research goal is to understand the underlying neurocognitive computations involved in different metacognitive judgments (Chua, Schacter & Sperling 2009; Metcalfe & Schwartz 2016; Shekhar & Rahnev 2024). To this end, research has focused on the factors that may facilitate or inhibit creation and efficiency of different metacognitive judgments, and several theories and computational models have been proposed (e.g., Brewer et al. 2010; Delay & Wixted 2021; Fleming & Daw 2017; Gollan & Brown 2006; Kiani, Corthell & Shadlen 2014; Koriat & Levy-Sadot 2001; Maniscalco et al. 2021; Maniscalco & Lau 2016; Metcalfe, Schwartz & Joaquim 1993; Pleskac & Busemeyer 2010; Schwartz & Metcalfe 2011; Shekhar & Rahnev 2021).

Despite considerable progress in the field of metacognition, there are still many critical unanswered questions. An important question, for example, is whether all metacognitive judgments are based on a general underlying neurocognitive mechanism(s) or whether each metacognitive judgment is the product of a unique neurocognitive processing (e.g., Barnes et al. 1999; Leonesio & Nelson 1990). This is a crucial question that has critical consequences for the scientific study of metacognition. For example, if all metacognitive judgments arise from a common neurocognitive mechanism(s), then we must ask how this mechanism(s) creates different metacognitive experiences, and if, indeed, these metacognitive experiences have different characteristics. On the other hand, if each metacognitive judgment is based on its unique mechanism(s), then one must ask whether there is an interaction between different mechanisms supporting different metacognitive judgments and how this interaction occurs.

In order to answer this question, we can adopt either or both of two general strategies, (1) correlation across judgment conditions and (2) the logic of dissociation. The first strategy is based on a simple assumption: if there is a correlation between the behavioral qualities or neural bases of a metacognitive judgment (e.g., the neural bases or metacognitive sensitivity^1^ of feeling-of-knowing) and the behavioral qualities or neural bases of the second metacognitive judgment (e.g., the neural bases or metacognitive sensitivity of post-decision confidence), then these two metacognitive judgments arise from a common neurocomputational mechanism(s). Utilizing this assumption, we can compare a metacognitive judgment when it is evaluating different cognitive/perceptual tasks (e.g., post-decision confidence evaluation of semantic vs. episodic memory recollection), or we can compare two different metacognitive judgments when they are evaluating the same cognitive/perceptual task (e.g., FOK and JOL evaluation of episodic memory recollection).

Studies using this strategy have found contrasting results. First, behavioral studies showed a correlation between evaluations of metacognitive sensitivity as well as post-decision confidence ratings for different cognitive/perceptual tasks within a modality (e.g., post-decision confidence evaluation of different perceptual decisions; see (Schraw et al. 1995; Schraw & Nietfeld 1998; Song et al. 2011; Veenman, Elshout & Meijer 1997), or across modalities (e.g., post-decision confidence evaluation of memory recollection and perceptual decisions; Faivre et al. 2018; McCurdy et al. 2013; Morales, Lau & Fleming 2018; but see Baird et al. 2013; Baird et al. 2015). Research, however, suggests that methodological differences between the two cognitive/perceptual tasks that a metacognitive judgment is evaluating may influence such behavioral correlations (Lee et al. 2018; Samaha & Postle 2017). Brain imaging studies, on the other hand, showed that distinct brain activities may underlie post-decision confidence evaluation of cognitive/perceptual tasks across modalities (Baird et al. 2013; Baird et al. 2015; McCurdy et al. 2013; Morales et al. 2018). Brian imaging studies also indicate that FOKs for semantic and episodic memory are based on different neurocognitive mechanisms (Elman et al. 2012; Imperio & Chua 2023; Reggev, Zuckerman & Maril 2011).

The second strategy, the dissociation strategy, seeks conditions in which there are changes in the qualities of one metacognitive judgment, while the second metacognitive judgment stays unaffected. For example, we can compare metacognitive sensitivity of FOK and post-decision confidence in a neurological condition (e.g., Alzheimer’s disease) and in a control group. If there is a change in metacognitive sensitivity of only one judgment (e.g., confidence), then these two metacognitive judgments are based on different neurocognitive mechanisms (for an application of this strategy in participants without a neurological condition, see Cleary 2015). On the other hand, if metacognitive sensitivity of both judgments changed in a comparable fashion, we can conclude that the two metacognitive judgments are based on a common neurocognitive mechanism(s).

The results of studies that adopted this strategy indicate that different metacognitive judgments are based on different neurocognitive mechanisms. For example, research showed lower metacognitive sensitivity of FOK in older adults than younger adults when FOK evaluated future episodic memory, but this age difference was not found in FOK evaluation of semantic memory recollection (Morson, Moulin & Souchay 2015; Souchay et al. 2007; but see Eakin, Hertzog & Harris 2014). Moreover, Souchay and Isingrini (2012) showed age-related differences in metacognitive sensitivity of FOK, but comparable metacognitive sensitivity of JOL between the two age-groups, suggesting a difference between the neurocognitive mechanisms underlying JOLs and FOK. In addition, Palmer, David, and Fleming (2014) reported age-related differences in metacognitive sensitivity of confidence, only when confidence was directed at perceptual judgments but not when directed at memory decisions (but see McWilliams et al. 2023). Further evidence in favor of the presence of distinct neurocognitive mechanisms for different metacognitive judgment comes from studies that evaluated metacognitive judgments in people who suffer from neurological conditions. For example, research suggests intact metacognitive sensitivity of confidence but impaired metacognitive sensitivity of FOK for people with Alzheimer’s disease (e.g., Pappas et al. 1992) and people with lesions to the medial prefrontal cortex (e.g., Schnyer et al. 2004; also see Pannu, Kaszniak & Rapcsak 2005).

Furthermore, behavioral research indicates that TOTs and FOKs are affected differently by working memory load (Schwartz 2008), even though some other factors may similarly impact both judgments (Metcalfe, Schwartz & Joaquim 1993; Schwartz et al. 2000; Yaniv & Meyer 1987), indicating that TOTs and FOKs might be based on different but interacting neurocognitive mechanisms. In line with this conclusion, a brain imaging study showed that TOT and FOK have unique but overlapping neural bases (Maril et al. 2005), and another report indicated that TOT and FOK are based on distinct brain activities (Huijbers et al. 2017). This leads to a conclusion that TOTs and FOK have some underlying mechanisms in common, but also some separate mechanisms as well. Hence, the results of the studies that adopted the dissociation strategy, suggest that different metacognitive judgments are based on different neurocognitive mechanisms.

In summary, research indicates that different metacognitive judgments are based on different but interacting neurocognitive mechanisms (also see Schwartz & Pournaghdali 2020). Support for the first part of this conclusion, that is the presence of distinct neurocognitive mechanisms for each metacognitive judgments, mainly comes from neuroimaging studies. Support for the second part of this conclusion, that is the presence of interaction between different metacognitive judgments and their neural underpinning, mainly comes from studies that showed correlation between behavioral qualities of different metacognitive judgments (e.g., FOK and post-decision confidence). Such behavioral evidence, however, is limited in explanatory value because it does not address the underlying nature of the interaction between different metacognitive judgments. Additionally, the methods used to estimate qualities of such metacognitive judgments (such as metacognitive sensitivity or metacognitive calibration) do not provide an exclusive estimate of such qualities. Hence, investigating the nature of the interaction between different metacognitive judgments seems to be of critical importance.

### Current Study

At least five decades of research have shown that TOTs and FOKs are associated with an increase in the possibility of retrieval of missing information (e.g., Bloom et al. 2018; Brown & McNeill 1966; Hertzog Dunlosky & Sinclair 2010; Koriat 1993; Koriat & Lieblich 1974; Metcalfe, Schwartz & Joaquim 1993; Sacher et al. 2009; Schwartz 2010; Schwartz, Pillot & Bacon 2014; Schwartz & Metcalfe 1992, but see Huebert, McNeely-White & Cleary 2023). Research also indicates that post-decision confidence is a good predictor of cognitive/perceptual decisions (e.g., Allen et al. 2017; Perfect 2002; Sinanaj, Cojan & Vuilleumier 2015; Yokoyama et al. 2010). Naturally, we can ask whether TOTs and FOKs can influence not only future memory recollection (see Cleary et al. 2021), but also the post-decision confidence evaluation of such recollection efforts. That is, will having a TOT or a high FOK influence later retrospective confidence, and will it change the accuracy of those judgments? Our question here is whether experience of TOT or high FOK may enhance the metacognitive sensitivity of post-decision confidence (henceforth, confidence sensitivity).

To answer this question, we employed a novel analytical approach based on a multidimensional extension of signal detection theory called general recognition theory (GRT; Ashby & Soto 2015; Ashby & Townsend 1986; Soto et al. 2017). In two experiments, we asked participants to provide TOT and FOK judgments on the trials that they were not able to recall the target information (e.g., the correct name of a celebrity in Experiment 1 and correct answer to a general knowledge question in Experiment 2). Next, we asked participants to perform a two-choice memory recognition task and to provide confidence ratings on the accuracy of their recognition decision (i.e., post-decision confidence). Finally, we fitted two GRT models, with TOT or FOK as one dimension of interest and confidence evaluation of memory recognition as the second dimension of interest (For a complete description of this model-based analysis, see section “Analysis”). Using the estimated parameters of the fitted models, we constructed two different type-2 sensitivity vs. metacognition curve (type-2 SvM curve). One model examined the influence of TOT on confidence sensitivity, and the other model examined the influence of FOK on confidence sensitivity. Each type-2 SvM curve represents changes in confidence sensitivity as a function of changes in the strength of internal evidence for TOT or FOK. Critically, by placing an objective criterion based on an optimal observer, we partitioned the x-axis of each type-2 SvM curve into two areas: the area of low likelihood of TOT or FOK, which is the area between zero and the objective criterion, and the area of high likelihood of TOT or FOK, which is the area to the right of the objective criterion.

Next, we examined the pattern of both type-2 SvM curves to evaluate the possible influence of TOT and FOK on confidence sensitivity. For both type-2 SvM curves, we expected one of two possible patterns, both involving an increase in confidence sensitivity as a function of the strength of TOT or FOK. That is, both patterns indicate that experience of TOT or FOK enhances confidence sensitivity. In the first pattern, confidence sensitivity deviates from the chance-level in the area of low likelihood of TOT or FOK, indicating the presence of residual and functional confidence sensitivity in the absence of TOT or FOK (P1). In the second pattern, confidence sensitivity deviates from the chance-level in the area of high likelihood of TOT or FOK, indicating the absence of residual and functional confidence sensitivity in the absence of TOT or FOK (P2).

Also possible is one of the following two patterns that are in contrast to our predictions: First, we may find a constant and horizontal type-2 SvM curve indicating the lack of association between a TOT or FOK and confidence sensitivity (P3). Second, we may find that confidence sensitivity decreases toward chance as the relative likelihood of TOT or FOK increases. This pattern indicates that experience of TOT or FOK suppresses post-decision confidence-related processing and sensitivity. To foreshadow, we found evidence for P1. That is, our results showed that confidence sensitivity deviates from the chance-level in the area of low likelihood of TOT and FOK and reaches its highest level in the area of high likelihood of TOT and FOK.

We also conducted a complementary analysis to provide a more intuitive understanding regarding the influence of TOT and FOK on post-decision confidence judgments. For this analysis, we were not concerned about sensitivity, that is, the predictive value of either TOTs or FOKs on the one hand, or post-decision confidence on the other. Rather, we were concerned if the presence of a TOT or a high FOK influences the magnitude of the post-decision confidence judgments. In this analysis, we estimated the mean post-decision confidence ratings for the trials that participants reported experiencing a TOT or a high FOK and compared each with the mean post-decision confidence ratings for the trial that participants reported not experiencing a TOT or a low FOK. We predicted higher post-decision confidence ratings in the trials that participants reported experiencing a TOT or a high FOK, similar to the results with the GRT approach. To foreshadow, the results confirmed this prediction.

## Experiment 1

The purpose of Experiment 1 was to evaluate the influence of TOT and FOK judgments on confidence sensitivity when using famous faces as cues. To this end, we presented photographs of famous people (activists, actors and actresses, athletes, musicians, politicians, etc.) and asked participants to recall their last names and then provide metacognitive judgments (TOTs and FOK) for each image for which they did not recall the name. Finally, participants performed a two-choice memory recognition task and rated their post-decision confidence on the accuracy of their recognition decision.

### Materials and Methods

#### Participants

A total of 76 undergraduate students were recruited from a pool of psychology undergraduate students at Florida Interactional University for one credit/hour compensation. Out of the 76 participants, 6 participants were removed from the final analysis, because they either recalled more than 80 percent of the correct names in the memory recall task (2 participants) or had less than 51 percent accuracy in the final memory recognition task (4 participants). Out of the remaining 70 participants, 64 participants identified as female, and their age ranged from 18 to 49. Each participant completed three behavioral sessions and received total of five credits. All of the participants had normal or corrected-to-normal vision and were able to read and write in English fluently. This experiment was conducted in accordance with the Declaration of Helsinki, and the Institutional Review Board of Florida International University approved it. Prior to the start of the experiment, participants consented to participate in this study.

#### Stimuli

We used a total of 400 photographs of famous people (200 male and 200 female) taken from the “Celebrity Face Recognition Dataset” (Mehta 2017). Each photograph was cropped in a way that only the face, part of the head, and the neck of the person were visible. We did this to eliminate any non-facial indicators of the name and fame of the faces. In addition, we resized each photograph to be more consistent in size. The final width of all of the photographs were 200 pixels but the height of photographs varied.

We also used 200 photographs of non-famous people (100 male and 100 female) for catch trails. We gathered these photographs by performing a Google image search. We only used images that have a “Creative Commons License”. Similar to the famous photographs, we cropped and resized non-famous photographs to eliminate any signs that may indicate that the people are not famous. In this experiment, we defined catch trials as the trials that are composed of photographs of non-famous people. Because these people were not famous, participants did not know their last names, and they should not have experienced a high level of a metacognitive experience (TOT and FOK) for such photographs, nor was there a known correct answer in recognition.

#### Procedure

This experiment consisted of 600 trials, which were completed in three online sessions, using the Qualtrics platform (fiu.qualtrics.com). At the beginning of each trial, we presented a photograph of a person that was either famous or non-famous and asked participants to recall the last name of that person. Participants were required to type the last name with uppercase letters. If participants did not know the last name, they could leave the answer space blank. Correct recall of a name resulted in termination of the trial and initiation of the next trial.

If memory recall failed for the name of the person in the photograph, we asked participants to perform the TOT and FOK tasks.^2^ In the TOT task, participants indicated if they were experiencing a TOT for the unrecalled item. This was a forced-choice task with two alternatives: “Yes” and “No”. In the FOK task, participants indicated if they had a high or low level of FOK. This was also a forced-choice task with two alternatives: “High” and “Low.” Participants were not able to see the images while performing the TOT and FOK tasks. Moreover, participants were required to perform these two tasks when recall failed.

After indicating the levels of TOT and FOK, we asked participants to perform a memory recognition task. In this task, we presented the target photograph again and provided participants with a last name that was either correct or incorrect. In half of the trials, we presented the correct last name, and in the other half of the trials, we presented an incorrect last name. Then, we asked participants to indicate if they thought that the provided last name was correct or incorrect using a forced-choice task with two alternatives: “Correct” and “Incorrect.” Regardless of the condition, in the memory recognition task, participants were required to choose one of the alternatives, even if they did not know the correct name. Finally, participants rated their confidence on their performance in the memory recognition task using one of the following alternatives: “very confident”, “somewhat confident”, “a little confident”, and “not at all confident.”

Each participant completed a total of three sessions with the maximum interval of 20 days between the first and the third sessions. At the beginning of each session, participants received instructions, which contained the definitions of TOT and FOK, and instructions for completing each task. To ensure that participants knew the exact instructions and definitions, we repeated the instructions in each session. Sessions one and two were composed of seven blocks each, and session three was composed of six blocks. Each block contained 30 trials: 10 correct name trials (trials with correct name condition), 10 incorrect name trials (incorrect name condition), and 10 catch trials. The order of trials within each block and the order of blocks within each session were randomized.

The order of which came first, the TOT task or the FOK task, was balanced across blocks to avoid order effects. To this end, the order of TOT and FOK was fixed within each block, but it changed from one block to the next. In session one, four blocks had TOT first and FOK second (TOT-first blocks), and three blocks had FOK first and TOT second (FOK-first blocks). In session two, four blocks were FOK-first blocks, and three were TOT-first blocks. And in session three, three blocks were TOT-first blocks, and three blocks were FOK-first blocks. Because we randomized the order of the blocks within each session, it is possible that a TOT-first block was followed by another TOT-first block. It is also possible that an FOK-first block was followed by another FOK-first block.

### Analysis

To evaluate the possible influence of TOTs and FOKs on confidence sensitivity, we performed two different analyses. Below is the description of each. For all of the analyses, we used R v. 3.6.3 extended with the package grtools v.0.3.1 (Soto et al. 2017; also see Pournaghdali et al. 2023). Mathematical details of the model and other aspects of the analysis are described in detail in the Supplementary Material.

#### GRT Analysis of Influence of TOT on Confidence Sensitivity

**Figure 1:**
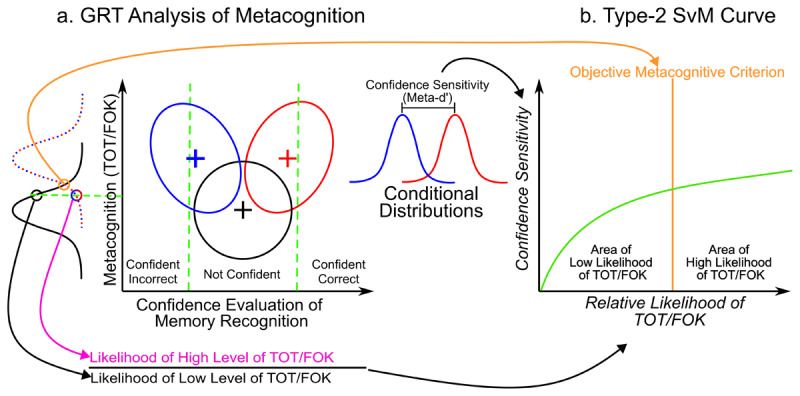
We first fitted the data to a GRT model, with TOT or FOK as one dimension of interest and post-decision confidence evaluation of memory recognition as the second dimension of interest **(a)**. Each ellipse in this figure represents a stimulus category (correct and incorrect name conditions as well as catch trials). Using the estimated parameters of the fitted models, we constructed a different type-2 SvM curve, which represents the influence of TOT or FOK on confidence sensitivity **(b)**.

First, we were interested in evaluating the association between TOT and confidence sensitivity. That is, our goal was to accurately determine the extent to which TOTs enhance confidence sensitivity. To this end, we fit a specific version of GRT called GRT with Individual Differences model (GRT-wIND model; Soto et al. 2015) to the behavioral data (see Figure 1a), using maximum likelihood estimation. Prior to fitting the GRT model, we reclassified participants’ post-decision confidence ratings as follow: “very confident” and “somewhat confident” responses were categorized as “Confident”, and “a little confident” and “not at all confident” were categorized as “Not Confident”. This was done to ensure that there would be enough data in each response category to allow for model fitting. Moreover, having two instead of four confidence levels allows us to formalize a more parsimonious model.

In this experiment, there were three stimulus conditions: correct name condition, incorrect name condition and catch trials. The y-axis of our GRT-wIND model represents the variation in the strength of internal evidence of each stimulus category along the TOT dimension. This axis also includes an objective criterion that an optimal metacognitive observer uses to maximize their correct categorization of their TOT experiences (see Pournaghdali et al. 2023; Pournaghdali et al. submitted). The x-axis represents post-decision confidence evaluation of memory recognition. More specifically, the x-axis represents the variation in the strength of internal evidence of each stimulus along the memory recognition dimension and includes two metacognitive criteria (two dotted green vertical lines in Figure 1a). One of these two criteria separates “confident incorrect” responses from “not-confident” responses, and the other criterion separates “not-confident” responses from “confident correct” responses.

Moreover, each stimulus condition (correct name, incorrect name and catch trials) is represented by a bidimensional normal distribution in the 2-dimensional decision space, depicted by an ellipse in Figure 1a. The bidimensional distribution that represents catch trials is depicted by an ellipse that is centered at zero in both x and y dimensions (black distribution). The other two stimuli are represented by two bidimensional distributions, higher in the y-axis and away from zero in opposite directions along the x-axis, corresponding to opposite memory recognition conditions (correct name in red and incorrect name in blue).

To make sure that the fitted model included the true maximum likelihood parameter estimates, we ran the optimization algorithm 100 times, each time starting from a different random configuration of starting parameters, and we kept the model with the highest likelihood. To avoid overfitting the GRT model and to address some concerns over the recoverability of variance parameters from GRT-wIND, we fixed the variance parameters to one (see Silbert & Thomas 2017). We also assumed that decision rules about TOT were not influenced by the post-decision confidence evaluation of memory recognition (i.e., decisional separability of TOT).

After fitting the GRT model to the behavioral data, we estimated two values for every point along the TOT dimension (e.g., the green dotted horizontal line in Figure 1a). The first value is the relative likelihood of TOT, which is the result of dividing the likelihood that a famous face was presented (i.e., the likelihood that true TOT was experienced) by the likelihood that a non-famous face (catch trial) was presented (i.e., the likelihood that no TOT should be experienced; Figure 1a). This parameter transfers the binary TOT judgments into a continuous experience with different strength levels (see Supplementary Material). The second value is the conditional confidence sensitivity (conditional meta-d’). Our conditional meta-d’, which is conceptually similar to meta-d’ proposed by Maniscalco and Lau (2012, 2014), is obtained by determining what d′ could explain the post-decision confidence data under the same distributional assumptions used to compute d′ from memory recognition data (Pournaghdali et al. 2023).

Next, we constructed a type-2 SvM curve, which represents changes in confidence sensitivity as a function of changes in the strength of TOT. The x-axis of the type-2 SvM curve is the relative likelihood of TOT, with zero representing the weakest level of TOT. As we move away from zero in this axis, the strength of TOT increases. The y-axis of the type-2 SvM curve is confidence sensitivity, with zero representing a point where meta-d’ is at chance. Moreover, by placing the objective criterion, we divide the x-axis of the type-2 SvM curve into two areas: the area of low likelihood of TOT, which is to the left of the objective criterion, and the area of high likelihood of TOT, which is to the right of the objective criterion.

**Figure 2:**
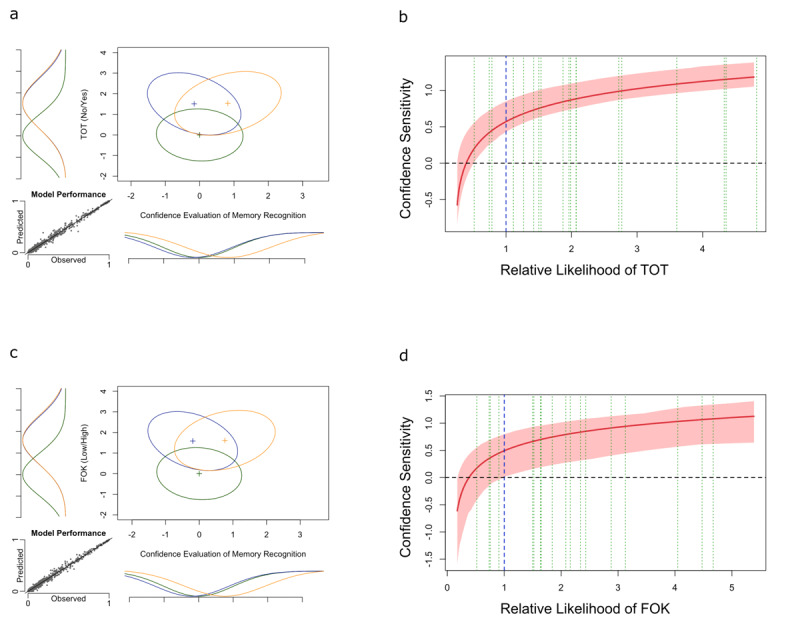
Experiment 1 results. The figures in the left column depict the estimated GRT models for each metacognitive judgment (TOT and FOK), and the figures in the right column depict estimated type-2 SvM curves. In all of the type-2 SvM curve graphs, the red line is the type-2 SvM curve extracted from the GRT model, and the lighter red bands represent the 99 percent confidence interval. The vertical blue line is the objective criterion based on the optimal observer in the GRT model, and the vertical green lines are the individual criterion of participants.

We were also interested in obtaining a 99% bootstrap confidence interval for the extracted type-2 SvM curve. To this end, we generated 1,000 simulated data samples from the fitted GRT model. Then, we fitted the model to each data sample again and obtained the type-2 SvM curve as indicated above. The result of this process is an empirical distribution function for type-2 SvM curves. We reported the simple percentiles from this function as limits for the 99% confidence interval, which is represented by the lighter red bands in Figure 2b and 2d.

#### GRT Analysis of Influence of FOK on Confidence Sensitivity

We were also interested in evaluating the association between FOK and confidence sensitivity. That is, our goal was to accurately determine the extent to which FOK may enhance confidence sensitivity. The analysis of influence of FOK on confidence sensitivity was the same as the previous analysis, with only one difference: The y-axis of the GRT model represents FOK and the x-axis of the SvM curve represents relative likelihood of FOK.

#### Evaluating the Influence of TOT on Average Post-Decision Confidence Rating

Next, we were interested in evaluating the influence of TOT experiences on the average post-decision confidence rating provided by participants. For this reason, we calculated each participant’s average post-decision confidence ratings in the trials that they reported experiencing a TOT (TOT trials) and the trials that they reported not experiencing a TOT (No-TOT trials). Then, we performed a Friedman test to compare average post-decision confidence rating between the TOT trials and the No-TOT trials. We chose the Friedman test because average post-decision confidence scores were not normally distributed (i.e., violation of normality).

#### Evaluating the Influence of FOK on Average Confidence Rating

Finally, we were interested in evaluating the influence of FOK on the average post-decision confidence rating provided by participants. For this reason, we calculated each participant’s average post-decision confidence ratings in the trials that they reported experiencing a high FOK (high-FOK trials) and the trials that they reported experiencing a low FOK (low-FOK trials). Then, we performed a Friedman test to compare average post-decision confidence rating between the high-FOK trials and the low-FOK trials.

### Results

Figure 2 presents the main results of Experiment 1. The figures in the left column (2a and 2c) depict the estimated GRT models for each metacognitive judgment (TOT and FOK), and the figures in the right column (2b and 2d) depict estimated type-2 SvM curves. In all of the type-2 SvM curve graphs, the red line is the type-2 SvM curve extracted from the GRT model, and the lighter red bands represent the 99 percent confidence interval. The vertical blue line is the objective criterion based on the optimal observer in the GRT model, and the vertical green lines are the individual criterion of participants.

#### Influence of TOT on Confidence Sensitivity

By accounting for 99.21% of variability in the behavioral data, the estimated GRT model provided a good fit to the data (Figure 2a). Figure 2b depicts the type-2 SvM curve representing confidence sensitivity as a function of strength of TOT. Based on this type-2 SvM curve, we think it is clear that confidence sensitivity is dependent on the strength of TOT experiences. That is, the confidence sensitivity is at the chance-level when participants are experiencing no-TOTs but is significantly above chance when TOTs occur. Accordingly, as the strength of TOT experiences increases, so does the confidence sensitivity. Furthermore, confidence sensitivity deviates from the chance-level in the area of low likelihood of TOT. This indicates the survival of post-decision confidence-related processes in the absence of a TOT experience.

#### Influence of FOK on Confidence Sensitivity

By accounting for 99.23% of variability in the behavioral data, the estimated GRT model provided a good fit to the data (Figure 2c). Figure 2d depicts the type-2 SvM curve representing confidence sensitivity as a function of strength of FOK. Based on this type-2 SvM curve, the confidence sensitivity is dependent on the strength of FOK experiences. That is, the confidence sensitivity is at the chance-level when participants are experiencing a low level of FOK but is significantly above chance at high levels of FOK. Accordingly, as the strength of FOK experiences increases, so does the confidence sensitivity. Furthermore, confidence sensitivity deviates from the chance-level in the area of low likelihood of FOK. This indicates the survival of post-decision confidence-related processes in the absence of an FOK experience.

#### Evaluating the Influence of TOT and FOK on Average Post-decision Confidence Rating

Evaluating the impact of TOT on average post-decision confidence rating showed that participants’ post-decision confidence ratings were significantly higher in the trials that they reported experiencing a TOT (*M* = 3.08, *SD* = 0.522) than the trials that they reported not experiencing a TOT (*M* = 1.48, *SD* = 0.415, χ2 (1) = 72, *p* < 0.001)). Evaluating the impact of FOK on average confidence rating showed that participants’ confidence ratings were significantly higher in the trials that they reported experiencing a high FOK (i.e., the trials that they selected the high FOK alternative; *M* = 2.94, *SD* = 0.485) than the trials that they reported experiencing a low FOK (i.e., the trials that they selected the low FOK alternative; *M* = 1.44, *SD* = 0.414, χ2 (1) = 72, *p* < 0.001). Hence, experiencing a TOT or a high FOK is associated with higher post-decision ratings of confidence.

### Discussion

The purpose of Experiment 1 was to evaluate the influence of TOT and FOK on confidence sensitivity when using famous faces as cues for recall of the person’s name. The results of the GRT-based analyses showed that experiencing a TOT or a high FOK is associated with increase in confidence sensitivity. We also found evidence for survival of post-decision confidence-related processes in the absence of a TOT or FOK experience. Finally, the results indicate that report of TOT or a high FOK is associated with higher post-decision confidence ratings. These results are true for metacognitive monitoring of semantic recollection for famous faces, but it remains to be seen if these results will generalize to other stimuli. Therefore, in the second experiment we replicated these results when participants recalled answers to a series of general-knowledge questions.

## Experiment 2

The purpose of Experiment 2 was to evaluate the influence of TOT and FOK judgments on confidence sensitivity when recollecting the answers to general-knowledge questions. To this end, we presented general-knowledge questions and asked participants to recall the answers to these questions and then provide metacognitive judgments (TOTs and FOK) for questions without correct recall. Finally, participants performed a two-choice memory recognition task and rated their post-decision confidence on the accuracy of their recognition decision.

### Materials and Methods

#### Participants

A total of 64 undergraduate students from Florida International University (FIU) participated in this experiment. We recruited the participants from a pool of psychology undergraduate students through the FIU Psychology Research Participants System (SONA) for one credit/hour compensation (each participant received 5 credits). Out of the 64 participants, 17 participants were removed from the final analysis, because they either recalled more than 80 percent of the correct answered in the memory recall task (4 participants) or had less than 51 percent accuracy in the final memory recognition task (13 participants). Out of the remaining 47 participants, 40 participants identified as female, and their age ranged from 18 to 40. Out of the remaining 40 participants, 40 participants identified as female, and their age ranged from 18 to 40. All of the participants had normal or corrected-to-normal vision and were able to read and write in English fluently. This experiment was conducted in accordance with the Declaration of Helsinki, and the Institutional Review Board of Florida International University approved it. Prior to the start of the experiment, participants consented to participate in this study.

#### Stimuli

The target stimuli were composed of 400 general-knowledge questions. Out of 400 questions, 274 questions were extracted from Tauber, Dunlosky, Rawson, Rhodes, and Sitzman (2013) updated norms based on the original Nelson and Narens (1980) norms. We created the remaining 126 questions (see the Supplementary Materials). We also created 200 questions with no correct answer (non-factual questions) for catch trials (see the Supplementary Materials). Because these questions had no correct answer (for example, what is the last name of the author of the novel “me and infinite worlds”?), participants should not know the answers to them, and they should not experience a high level of metacognition (TOT and high FOK) for such questions. We removed one of the non-factual questions from the final analysis, because depending on the context, the question could have a correct response (What is the first name of Mrs. Potts’ husband in the movie Beauty and the Beast?).^3^

#### Behavioral Tasks, Procedure, and analyses

The behavioral tasks, procedure and analyses were identical to those of Experiment 1.

### Results

**Figure 3:**
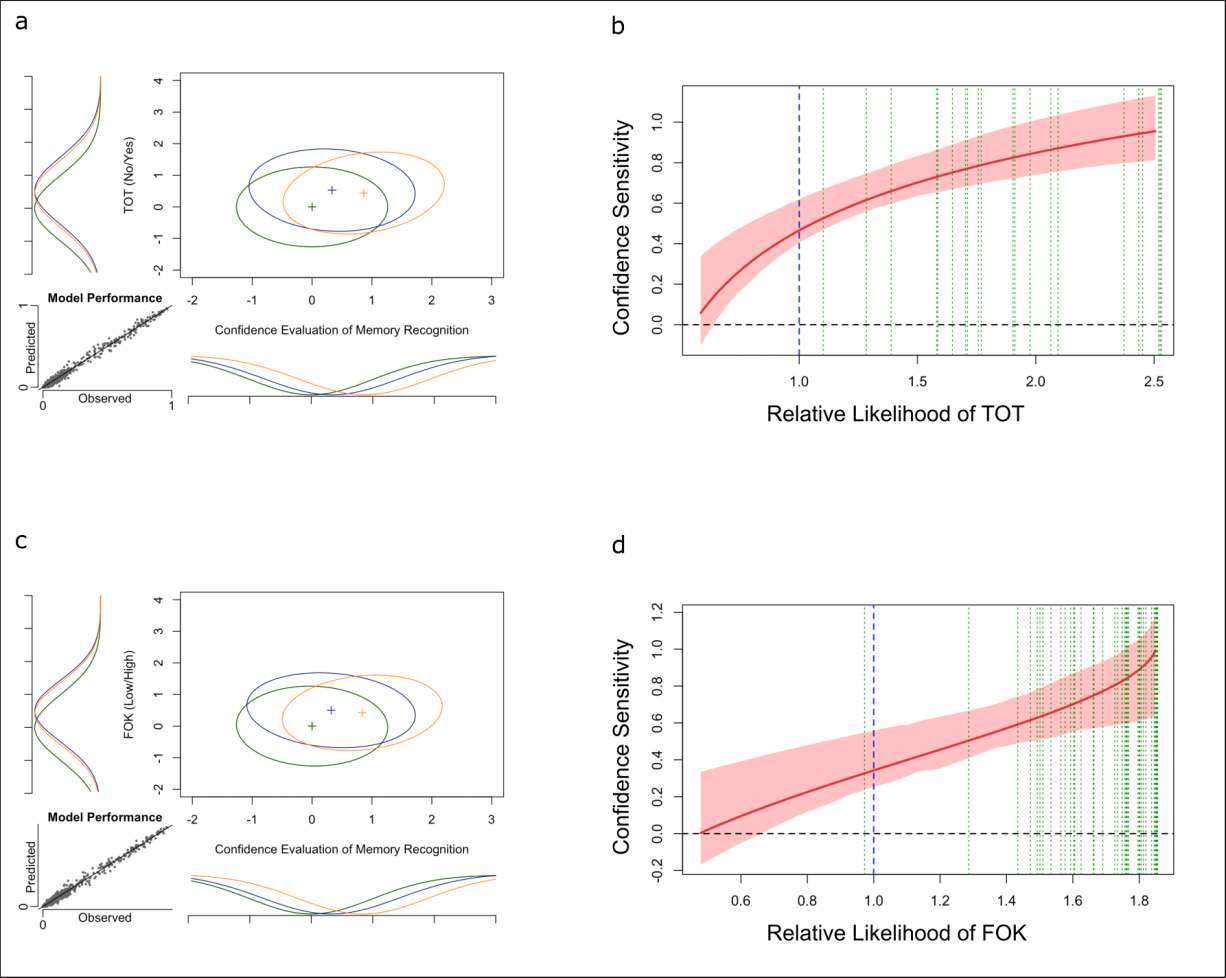
Experiment 2 results. The figures in the left column depict the estimated GRT models for each metacognitive judgment (TOT and FOK), and the figures in the right column depict estimated type-2 SvM curves. In all of the type-2 SvM curve graphs, the red line is the type-2 SvM curve extracted from the GRT model, and the lighter red bands represent the 99 percent confidence interval. The vertical blue line is the objective criterion based on the optimal observer in the GRT model, and the vertical green lines are the individual criterion of participants.

Figure 3 presents the main results of Experiment 2. The structure and interpretation of this figure is the same as the description of Figure 2.

#### Influence of TOT on Confidence Sensitivity

By accounting for 98.89% of variability in the behavioral data, the estimated GRT model provided a good fit to the data (Figure 3a). Figure 3b depicts the type-2 SvM curve representing confidence sensitivity as a function of strength of TOT. Based on this type-2 SvM curve, the confidence sensitivity is dependent on the strength of TOT experiences. That is, the confidence sensitivity is at the chance-level when participants are experiencing a no-TOT but is significantly above chance when TOTs occur. Accordingly, as the strength of TOT experiences increases, so does the confidence sensitivity. Furthermore, confidence sensitivity deviates from the chance-level in the area of low likelihood of TOT. This indicates the survival of post-decision confidence-related processes in the absence of a TOT experience.

#### Influence of FOK on Confidence Sensitivity

By accounting for 98.86% of variability in the behavioral data, the estimated GRT model provided a good fit to the data (Figure 3c). Figure 3d depicts the type-2 SvM curve representing confidence sensitivity as a function of strength of FOK. Based on this type-2 SvM curve, the confidence sensitivity is dependent on the strength of FOK experiences. That is, the confidence sensitivity is at the chance-level when participants are experiencing a low level of FOK but is significantly above chance at high levels of FOK. Accordingly, as the strength of FOK experiences increases, so does the confidence sensitivity. Furthermore, confidence sensitivity deviates from the chance-level in the area of low likelihood of FOK. This indicates the survival of post-decision confidence-related processes in the absence of an FOK experience.

#### Evaluating the Influence of TOT and FOK on Average Post-decision Confidence Rating

Evaluating the impact of TOT on average post-decision confidence ratings showed that participants’ post-decision confidence ratings were significantly higher in the trials that they reported experiencing a TOT (*M* = 2.90, *SD* = 0.523) than the trials that they reported not experiencing a TOT (*M* = 1.76, *SD* = 0.509, χ2 (1) = 47.1, *p* < 0.001). Evaluating the impact of FOK on average confidence ratings showed that participants’ post-decision confidence ratings were significantly higher in the trials that they reported experiencing a high FOK (i.e., the trials that they selected the high FOK alternative; *M* = 2.88, *SD* = 0.454) than the trials that they reported experiencing a low FOK (i.e., the trials that they selected the low FOK alternative; *M* = 1.66, *SD* = 0.412, χ2 (1) = 51, *p* < 0.001). Hence, experiencing a TOT or a high FOK is associated with higher post-decision ratings of confidence.

### Discussion

The purpose of Experiment 2 was to evaluate the influence of TOT and FOK on confidence sensitivity when using general-knowledge questions. The results of the GRT-based analyses showed that experiencing a TOT or a high FOK is associated with increase in confidence sensitivity. We also found evidence for survival of post-decision confidence-related processes in the absence of a TOT or FOK experience. Finally, the results indicate that report of TOT or a high FOK is associated with higher post-decision confidence ratings.

## General Discussion

The main goal of this study was to evaluate the possible influence of TOT and FOK on confidence sensitivity. That is, we were interested in determining whether experiencing a TOT or a high FOK may be associated with increase in the metacognitive sensitivity of post-decision confidence judgments. In two experiments, we utilized GRT-based analysis and constructed two separate type-2 SvM curves: one to examine the influence of TOT on confidence sensitivity and one to examine the influence of FOK on confidence sensitivity. The results of both experiments indicated that post-decision confidence judgments were more indicative of memory recognition performance when participants were experiencing a TOT or a high FOK. That is, the accuracy of post-decision confidence judgments improved when people had experienced a TOT or a high FOK earlier in the trial. To the best of our knowledge, this is the first report of the influence of TOT and FOK on confidence sensitivity. Moreover, in both experiments TOTs and high FOKs were also associated with higher post-decision confidence ratings.

It should be noted that for both tasks, we used a binary scale because of model-fitting concerns. We, however, used different response alternatives across the two tasks: yes/no for TOT and high/low for FOK. The main reason for the difference in alternatives between the two tasks is the ease of understanding the task and using the response options for our participants. It seems that high/low FOK is easier to understand for participants than yes/no FOK. For the TOT, on the other hand, the yes/no binary option is easier to understand and use. Moreover, we tried to keep our procedure and tasks as similar as possible to those of other studies. Therefore, we only changed parts of the methodology that were necessary for our model-based analysis. Even though we used binary scales for both the TOT and FOK tasks, we kept elements of the tasks similar to other studies, namely the all-or-none nature of a TOT and the steps for FOK. Regardless of the scales used, we argue that the choice of response options does not influence the model-fitting processes and the final results.

The results of the type-2 SvM analyses as well as the results indicating influence of report of TOT and FOK on the average post-decision confidence ratings are in line with a hierarchical conceptualization of metacognition (Arango-Muñoz 2011). According to this conceptualization, the metacognitive system is composed of two levels: the low-level metacognition that is composed of fast, automatic, and nonconscious metacognitive processes and the high-level metacognition that is composed of slow and conscious metacognitive experiences (also see Koriat 2015). Following this conceptualization, we can classify TOTs and FOKs as high-level metacognitive experiences, as they are conscious metacognitive experiences (see Schwartz & Pournaghdali 2016; Schwartz & Pournaghdali 2023). Post-decision confidence-related processing, on the other hand, can operate in the presence and absence of consciousness (see Jachs et al. 2015; Pournaghdali et al. 2023). Hence, depending on the presence or absence of consciousness, post-decision confidence can be a high or a low-level metacognitive experience (see below).

Based on this, we argue that TOT and FOK impact the confidence sensitivity based on four possibilities that may not be mutually exclusive. First, it is possible that experience of TOT and FOK have a top-down influence on memory processes. Hence, by enhancing the quality of the memory information available to the [post-decision] confidence system and making such information more salient, TOT and FOK may enhance post-decision confidence-related processes. Indeed, recent evidence indicates that when people are in TOTs, they seek more information and work harder to retrieve information (Huebert et al. 2023; Metcalfe, Schwartz & Bloom 2017; for similar results with post-decision confidence judgments, see Metcalfe et al. 2023). Second, it is possible that TOT and FOK have a direct top-down influence on post-decision confidence itself, regardless of their top-down influence on memory processes. That is, the enhancement of confidence sensitivity might be the result of direct top-down influence of TOT and FOK on post-decision confidence-related processes. Based on this possibility, experience of TOT and FOK may improve efficiency of post-decision confidence-related process in evaluating object-level information. In this view, a sharp metacognitive experience, such as TOT or high FOK, primes post-decision confidence-related processes to be more sensitive to memory processes, leading to an enhanced confidence sensitivity as well as a higher post-decision confidence rating when participants experience a TOT or a high FOK. Supporting the first two possibilities comes from a study showing that reporting a TOT leads to a higher chance of recognition without identification, which is the feeling that participants have seen the correct response before (Cleary 2006). Similar to our results, the experience of TOT may have a top-down influence on memory and/or confidence-related processes. Third, it is possible that there are two modes of post-decision confidence experiences: a low-level post-decision confidence that provides a coarse evaluation of object-level processes and a high-level post-decision confidence that provides a finer evaluation of object-level processes. Based on this, it is possible that TOT and FOK trigger the high-level post-decision confidence processes, and in this way, enhance the confidence sensitivity. Finally, it is also possible that a common metacognitive mechanism exists. In this case, TOT, FOK, and post-decision confidence are different elements of this metacognitive system. Accordingly, enhancement of a general metacognitive evaluation of object-level processes may manifest itself as simultaneous enhancement of metacognitive sensitivity of TOT, FOK and post-decision confidence. However, the above chance-level confidence sensitivity in the area of low likelihood of TOT and FOK argue against this possibility. If there was only one metacognitive system with different elements, then we would expect chance-level confidence sensitivity in the absence of TOT and FOK judgments. We argue that future research on metacognition should evaluate all of these possibilities. To summarize our conclusions, we found that already having a strong metacognitive experience, such as a high FOK or a TOT, increases metacognitive sensitivity of a subsequent metacognitive judgment, that is, the post-decision confidence judgments given after recognition in this study.

## Data Accessibility Statement

The specific hypotheses tested in this manuscript were not preregistered. The model-fitting procedures, estimated models, behavioral data, and R syntax used to perform the analyses are available at https://osf.io/7r9hd/.

## Additional File

The additional file for this article can be found as follows:

- **Supplementary Material** https://osf.io/7r9hd/.

## Footnotes

We define metacognitive sensitivity as the ability of a metacognitive judgment to accurately predict cognitive/perceptual sensitivity or the association between a metacognitive experience and enhancement of a cognitive/perceptual sensitivity (See Pournaghdali et al., submitted).

In some trials, participants might have misspelled a correct response (e.g., McConaghey instead of McConaughey). The program considered such trials as incorrect and asked participants to perform TOT, FOK, recognition and post-decision confidence tasks. To resolve this issue, author AP returned to the data later and removed TOT, FOK, recognition and post-decision confidence responses of such trials and coded them as correct.

In live action adaptation of Beauty and the Beast (2017), Jean Potts was husband of Mrs. Potts. This character, however, was not part of the animated Beauty and the Beast (1991).
